# Therapeutic Anticoagulation Delays Death in COVID-19 Patients: Cross-Sectional Analysis of a Prospective Cohort

**DOI:** 10.1055/s-0040-1716721

**Published:** 2020-09-26

**Authors:** Filip Ionescu, Giovi Grasso-Knight, Edward Castillo, Ehsun Naeem, Ioana Petrescu, Zaid Imam, Vishal K. Patel, Mangala Narasimhan, Girish B. Nair

**Affiliations:** 1 Department of Internal Medicine, Beaumont Health System, Royal Oak, Oakland University William Beaumont School of Medicine, Michigan, United States; 2 Division of Pulmonary and Critical Care Medicine, Beaumont Health System, Royal Oak, Oakland University William Beaumont School of Medicine, Michigan, United States; 3 Department of Radiation Oncology, Beaumont Health System, Royal Oak, Oakland University William Beaumont School of Medicine, Michigan, United States; 4 Department of Computational and Applied Mathematics, Rice University, Texas, United States; 5 Division of Pulmonary and Critical Care Medicine, North Well, New York, New York, United States

**Keywords:** anticoagulation, COVID-19, novel coronavirus, heparin

## Abstract

A hypercoagulable state has been described in coronavirus disease 2019 (COVID-19) patients. Others have reported a survival advantage with prophylactic anticoagulation (pAC) and therapeutic anticoagulation (tAC), but these retrospective analyses have important limitations such as confounding by indication. We studied the impact of tAC and pAC compared with no anticoagulation (AC) on time to death in COVID-19. We performed a cross-sectional analysis of 127 deceased COVID-19 patients and compared time to death in those who received tAC ( *n* = 67), pAC ( *n* = 47), and no AC ( *n* = 13). Median time to death was longer with higher doses of AC (11 days for tAC, 8 days for pAC, and 4 days for no AC, *p* < 0.001). In multivariate analysis, AC was associated with longer time to death, both at prophylactic (hazard ratio [HR] = 0.29; 95% confidence interval [CI]: 0.15 to 0.58; *p* < 0.001) and therapeutic doses (HR = 0.15; 95% CI: 0.07 to 0.32; *p* < 0.001) compared with no AC. Bleeding rates were similar among tAC and remaining patients (19 vs. 18%; *p* = 0.877). In deceased COVID-19 patients, AC was associated with a delay in death in a dose-dependent manner. Randomized trials are required to prospectively investigate the benefit and safety of higher doses of AC in this population.

## Introduction

Severe acute respiratory syndrome-coronavirus-2 (SARS-CoV-2) is a novel coronavirus identified as the cause of several cases of severe pneumonia in December 2019 in Wuhan, China, later designated as novel coronavirus disease 2019 (COVID-19) by the World Health Organization (WHO). 1 2 It has since spread exponentially to become a global pandemic. 3 4 An increasing body of evidence suggests hypercoagulability as an important component in the pathogenesis of severe COVID-19. Bedside reports of frequent clotting of central venous and hemodialysis catheters have now been supplemented by laboratory data consistent with activation of the coagulation cascade as quantified by elevated D-dimer and fibrinogen in conjunction with low antithrombin levels. 5 The prevalence of disseminated intravascular coagulation (DIC) per International Society for Thrombosis and Haemostasis (ISTH) criteria was markedly higher in deceased patients compared with survivors (71 vs. 0.6%). 6 Two studies employing thromboelastography have shown a coagulation profile consistent with hypercoagulability in the context of severe systemic inflammation. 7 8 That hypercoagulability is of particular clinical importance is also supported by multiple autopsy case series reporting pulmonary and other visceral microthromboses. 9 10 11

A case series of thrombotic complications in critically ill patients with COVID-19 noted a high incidence of 31%, with pulmonary embolism as the most frequent manifestation. 12 Furthermore, one retrospective study found prophylactic anticoagulation (pAC) to be associated with lower mortality at 28 days in certain subgroups of patients with severe disease and demonstrated hypercoagulable profile either by a sepsis-induced coagulopathy (SIC) score ≥4 or D-dimer >6-fold the upper limit of normal. 13 A more recent retrospective analysis reported a possible survival advantage for severe COVID-19 patients treated with therapeutic AC, especially in a mechanically ventilated subgroup; the effect observed was duration dependent. 14

These observations prompted several professional societies to publish consensus statements on management of COVID-19-associated coagulopathy. 4 15 16 All recommend obtaining initial standard coagulation tests, D-dimer and fibrinogen levels, frequent retesting, and continuing pAC throughout the hospitalization with possible extension beyond discharge for patients at higher risk of venous thromboembolism. Individual papers have also discussed using higher doses of anticoagulation (AC) 12 or even tissue plasminogen activator, 17 both for prevention of thrombotic disease, and for the anti-inflammatory effect of heparin. 18

Data regarding the outcomes of severe COVID-19 patients treated with therapeutic AC (tAC) is scarce and this treatment modality has not been incorporated in guidance from expert panels. Information on both the efficacy and the safety of tAC is urgently needed. We aimed to report the impact of tAC on time to death based on cross-sectional analysis of a prospective cohort followed-up at our institution, where criteria for initiating tAC have been adopted.

## Methods

### Study Design

A single-center, cross-sectional analysis of deceased patients included in a prospective cohort was conducted at William Beaumont Hospital (Royal Oak, Michigan, United States), a tertiary care academic teaching hospital. Patients aged 18 years or older who tested positive for SARS-CoV-2 on nucleic amplification testing (NAAT) of nasopharyngeal secretions over the first 4 weeks of the COVID-19 pandemic in Michigan (March 13–April 8, 2020), and who expired from related complications were identified by active surveillance of inpatient records. At the time of publication, the majority of COVID-19 patients remain hospitalized at our institution and the final course of their illness is uncertain. We chose to focus on a cohort of deceased patients with unequivocal outcome, which has the added advantage of avoiding confounding by indication. Our institution has adopted criteria for initiating tAC, which include the presence of worsening respiratory failure, impending or actual need for mechanical ventilation, and worsening kidney failure, and/or a D-dimer > 6-fold the upper limit of normal (>3,000 ng/mL FEU). By consensus, the recommended duration of AC is limited to 5 days, with extension beyond this timeframe if there is a clear indication for continuing AC or the treating clinician chooses to continue. The study was approved by Institutional Review Board (IRB no.: 2020–125).

Patient demographics (age, sex, and ethnicity), laboratory data, and information about therapeutic modalities (AC, corticosteroids [CS], vasopressor, etc.) were obtained from review of electronic medical records prospectively on a daily basis, using an IRB-approved data collection checklist. Therapeutic AC (tAC) was defined as use of unfractionated heparin (UFH) as an intravenous infusion with documented activated partial thromboplastin time (aPTT) in the AC range (≥45 seconds), subcutaneous enoxaparin at doses of 1 mg/kg twice daily or 1.5 mg/kg once daily (while allowing for dose adjustment based on creatinine clearance), or oral AC prescribed for a preexisting established indication in the form of warfarin with documented therapeutic international normalized ratio (2–4) or direct oral anticoagulants (apixaban and rivaroxaban). The pAC was defined as subcutaneous injection of UFH at doses of 5,000 units twice or three daily, or subcutaneous enoxaparin injection at doses of 30 to 40 mg once daily. Immunosuppressive CS therapy was defined as at least one dose of 15 mg methylprednisolone or equivalent dose of other CS.

Major bleeding was defined according to the ISTH definition as having a symptomatic presentation and (1) fatal bleeding; and/or (2) bleeding in a critical area or organ such as intracranial, intraspinal, intraocular, retroperitoneal, intra-articular or pericardial, or intramuscular with compartment syndrome; and/or (3) bleeding causing a fall in hemoglobin level of 20 g L ^−1^ (1.24 mmol L ^−1^ ) or more, or leading to transfusion of two or more units of whole blood or red cells. 19 Active cancer was also defined according to ISTH as cancer diagnosed within the previous 6 months, recurrent, regionally advanced or metastatic cancer, cancer for which treatment had been administered within 6 months, or hematologic cancer that was not in complete remission.

### Outcomes and Statistical Analysis

The main outcome was the time to death of COVID-19 patients compared between patients who received tAC, pAC, and no AC. Time zero was the time of admission for patients admitted for COVID-19 and the time of positive NAAT for SARS-CoV-2 for those who developed symptoms during the hospitalization. A multivariate Cox proportional hazards model was performed to assess the impact of candidate variables on the retrieval rate. Candidate variables were preliminarily tested for significance in univariate Cox proportional hazards model using backward and forward regression.

Statistical analysis was performed using JMP version 14 and SAS 9.4 (SAS Institute, Cary, North Carolina, United States). Categorical variables are described as frequency (percentage). Normal or approximately normal variables are reported using the mean (±standard deviation), whereas skewed variables are reported with the median (interquartile range [IQR]). Categorical variables were compared using the Chi-square test or Fisher's exact test. Normal variables were compared using a two-sided Student's *t* -test and ordinal variables used the Kruskal–Wallis test. All *p* -values were two-sided and a *p* < 0.05 was considered to indicate statistical significance.

## Results

**Fig. 1:**
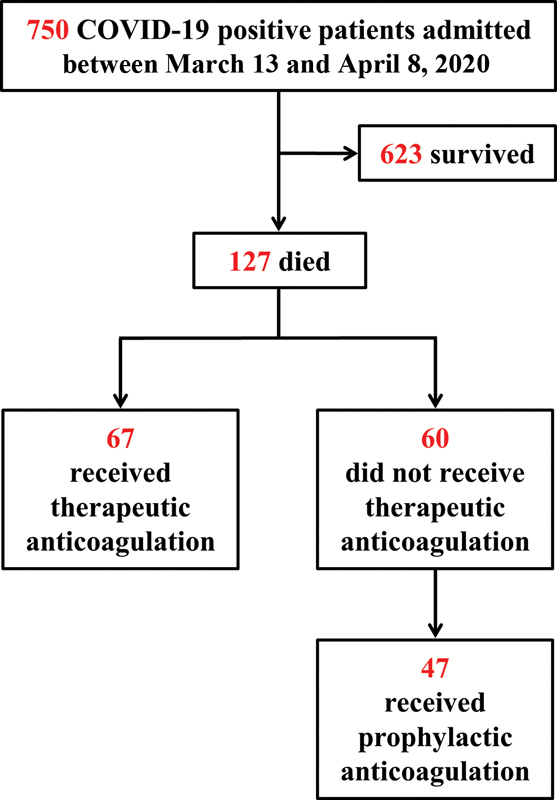
Study population. COVID-19, novel coronavirus disease 2019.

Over 30 days (March 13–April 8, 2020), 750 patients diagnosed with COVID-19 were admitted and there were 127 (17%) COVID-19-related deaths. Fig. 1 provides an outline of the study population. The mean age was 74 years (±15) and 68 (54%) patients were male. Racial distribution was notable for a predominance of African Americans (71, 56%); 51 were Caucasian (40%) and 2 (2%) were Southeast Asian. Information about ethnicity was not available for two patients.

### Baseline Characteristics. Treatment with Anticoagulation

Sixty-seven (53%) patients received tAC with intravenous UFH employed in 87%; for 3% treatment was with subcutaneous enoxaparin, whereas 10% of patients were continued on home oral anticoagulants (7% direct oral anticoagulants and 3% warfarin). None of the patients were receiving pAC upon admission. Of the remaining 60 patients who did not receive tAC, 47 (37%) received pAC, while only 13 (10%) did not receive any ACs. The baseline characteristics of patients are shown in Table 1 . The median duration of tAC was 5 days (IQR: 3–8 days) and it was initiated on median day 6 of hospitalization (IQR: 2–9 days). The number of days on tAC did not significantly differ between patients who were treated in the intensive care unit (ICU) and those who were not (median = 5 days [IQR: 3–10 days] vs. 4.5 days [IQR: 2–6 days]; *p* = 0.267). For most patients (37, 55%), tAC was initiated empirically for hypercoagulability related to COVID-19. Atrial fibrillation accounted for the majority of remaining indications (21, 31%), followed by chronic venous thromboembolism (5, 7%), acute arterial thromboembolism (2, 3%), and acute coronary syndromes (2, 3%). In our analysis, one patient experienced arterial embolism and one was diagnosed with incidental aortic thrombus during their hospitalization for COVID-19. It is important to note that, due to institutional policy aimed at preventing viral spread, the number of diagnostic imaging obtained was significantly limited: only seven (6%) patients had venous Doppler ultrasound to investigate for deep vein thrombosis (three were positive) and only three (2%) had computed tomography of the chest with intravenous contrast to diagnose pulmonary embolism (one was positive).

**Table 1:** Baseline characteristics and comorbid conditions in study population

	All patients ( *n* = 127)	Therapeutic anticoagulation ( *n* = 67)	Not on therapeutic anticoagulation ( *n* = 60)	Significance
Age in years (SD)	74 (15)	72 (12)	77 (18)	0.051
Male sex	68 (54)	36 (54)	32 (53)	0.964
Caucasian race	51 (40)	21 (31)	30 (50)	0.032
BMI > 30 kg/m ^2^	53 (42)	39 (58)	14 (23)	<0.0001
Diabetes	59 (46)	34 (51)	25 (42)	0.305
Hypertension	96 (76)	52 (78)	44 (73)	0.575
Coronary artery disease	37 (29)	17 (25)	20 (33)	0.324
Heart failure	27 (21)	16 (24)	11 (18)	0.444
VTE	14 (12)	9 (14)	5 (9)	0.407
Atrial fibrillation	12 (10)	9 (14)	3 (5)	0.134
Ischemic stroke or TIA	19 (16)	13 (21)	6 (11)	0.209
AKI a on admission	63 (50)	27 (53)	36 (75)	0.022
CKD grade 3 and above	34 (27)	15 (22)	19 (32)	0.238
Hemodialysis-dependent	9 (7)	3 (4)	6 (10)	0.226
Chronic lung disease	29 (23)	16 (24)	13 (22)	0.766
Active cancer	8 (6)	4 (6)	4 (7)	0.871
Ever smoker	63 (50)	32 (48)	31 (52)	0.660
ICU stay	75 (59)	55 (82)	20 (33)	<0.0001
Corticosteroid treatment	65 (51)	43 (64)	22 (37)	0.002

Abbreviations: AKI, acute kidney injury; BMI, body mass index; CKD, chronic kidney disease; ICU, intensive care unit; SD, standard deviation; TIA, transient ischemic attack; VTE, venous thromboembolism.

Note: Age is presented as mean (standard deviation). Other numbers presented as *n* (%).

a Defined by an elevation in serum creatinine of 0.3 mg/dL or more relative to known baseline value (data available for 99 patients).

Overall, the tAC group was younger (72 vs. 77 years; difference 5.3 years; 95% confidence interval [CI], −0.03 to 10.7 years; *p* = 0.051), had a higher frequency of obesity (58 vs. 23%; *p* < 0.0001) and had fewer Caucasian patients (31 vs. 50%; *p* = 0.032). Patients who received tAC were also more frequently treated in an ICU (82 vs. 33%; *p* < 0.0001). Peak D-dimer levels recorded throughout admission were significantly higher in the tAC group (median = 10,000 ng/mL [IQR: 4,195–10,000 ng/mL] versus median of 2,230 ng/mL [IQR: 1,453–6,698 ng/mL]; *p* = 0.001). By contrast, peak fibrinogen measurements were similar in both groups (672 [±191] vs. 587 [±209] mg/dL; *p* = 0.090).

Patients who did not receive tAC had a higher frequency of acute kidney injury defined as elevation in serum creatinine of >0.3 mg/dL compared with known baseline (75 vs. 53%; *p* = 0.022). For the 118 patients who were not dialysis dependent prior to admission, 27 (42%) in the tAC group compared with 6 (11%) in the no AC group were provided renal replacement therapy during their hospitalization ( *p* < 0.001).

### Treatment with Corticosteroids

A total of 65 (51%) patients were treated with immunosuppressive doses of CS. Treatment was initiated on a median day 4 (IQR: day 2–7) of hospitalization and the median duration of treatment was 5 (IQR: 3–7) days. CS treatment was more prevalent in the tAC group (64 vs. 37%; *p* = 0.002) and was administered for a longer duration (median = 6 days [IQR: 5–7 days] vs. 4 days [IQR: 2–5 days]; *p* < 0.001), but it was initiated later in the hospital course (median day 4 of admission [IQR: day 2–8] vs. day 2 [IQR: day 1–4]; *p* = 0.013). The most commonly used CS formulation was intravenous methylprednisolone at doses between 40 and 90 mg daily (77% of patients received 40 mg twice daily).

### Patients Treated in the Intensive Care Unit

Seventy-five patients were treated in the ICU and most required intubation with mechanical ventilation (68, 91%) and vasopressor support (63, 84%). The mean time from admission to intubation was 5 (±3) days and the median duration of mechanical ventilation was 7 days (IQR: 4–9 days). Approximately one-third of patients (24, 32%) required paralytics to ensure optimal ventilation and one quarter underwent prone ventilation (17, 23%). Nearly half of ICU patients (32, 43%) required initiation of continuous renal replacement therapy or intermittent hemodialysis.

### Complications to Specific Therapies

Bleeding rates were similar between the tAC and non-AC groups. Estimates included bleeding of any severity (19 vs. 18%; *p* = 0.877), any bleeding requiring transfusion (7 vs. 8%; *p* = 0.855), and major bleeding (3 vs. 8%; *p* = 0.18).

The rate of bacterial and fungal superinfections was estimated using the results of microbiological studies. Blood cultures were obtained in 110 (87%) and respiratory cultures in 85 (67%) patients. Overall, patients who received CS had a higher frequency of positive blood (18 vs. 8%; *p* = 0.116) and respiratory cultures (13 vs. 8%; *p* = 0.510), but these differences did not reach statistical significance.

### Impact of Therapeutic Anticoagulation and other Patient Variables on Time to Death

The median time from admission to death in the entire population was 9 days. In addition to patient factors which differed significantly in group comparison (age, Caucasian race, body mass index [BMI] > 30 kg/m ^2^ , ICU stay, and CS treatment), Cox regression identified additional candidate variables. Those which predicted an increase in time to death were: hypertension ( *p* = 0.21), CS treatment (HR = 0.56; *p* = 0.001), and duration of CS treatment ( *p* < 0.001). Ever smoker status was associated with a shorter time to death ( *p* = 0.019). Other demographics or comorbid conditions (sex, diabetes, heart failure, etc.) did not exhibit a significant relation (defined as *p* < 0.25) with time to death in univariate analysis.

**Fig. 2:**
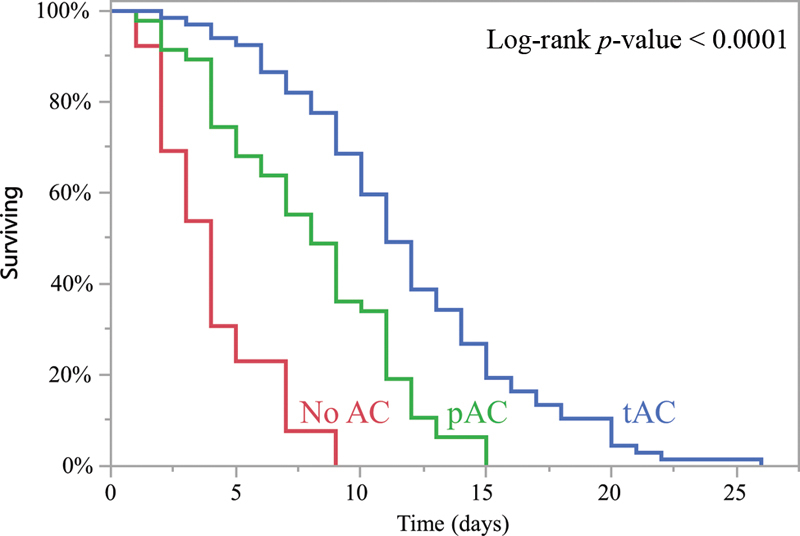
Kaplan–Meier analysis of time to death in patients who received therapeutic (tAC), prophylactic (pAC) or no anticoagulation (AC).

**Fig. 3:**
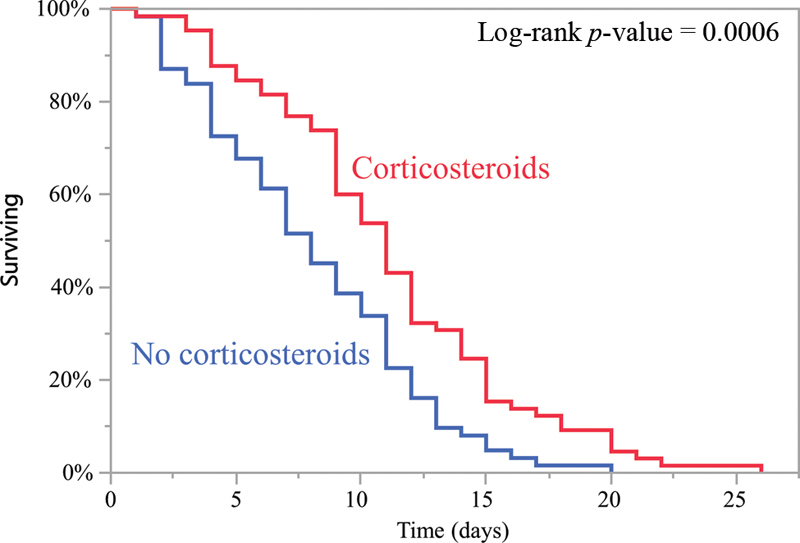
Kaplan–Meier analysis of time to death in patients who received immunosuppressive doses of corticosteroids and those who did not.

The Kaplan–Meier curve comparing time to death for patients who received AC at different doses or no AC is presented in Fig. 2 . In univariate analysis, death was increasingly delayed with increasing doses of AC: median of 11 days for tAC, 8 days for pAC, and 4 days for no AC ( *p* < 0.001). Fig. 3 compares time to death for patients treated with CS and for those who were not (median: 11 vs. 8 days; *p* = 0.001).

Results of the multivariate Cox proportional hazards model including the remaining significant variables after backward and forward regression are summarized in Table 2 . The final model found that AC was an independent predictor of longer time to death, both when administered at prophylactic doses (HR = 0.29; 95% CI: 0.15–0.58; *p* < 0.001) and at therapeutic doses (HR = 0.15; 95% CI: 0.07–0.32; *p* < 0.001) compared with no AC. The duration of CS treatment was similarly associated with increased time to death (HR = 0.89 per 1-day increase; 95% CI: 0.84–0.93; *p* < 0.001). Ever smoker status, by contrast, predicted a shorter time to death (HR = 1.86; 95% CI: 1.25–2.8; *p* = 0.002). None of the other suggested variables showed an impact on time to death in the multiple regression model.

**Table 2:** Multivariate Cox proportional hazards model (all patients, *n* = 127)

	Hazard ratio	Confidence interval	Significance
Ever smoker	1.86	1.25–2.8	0.002
CKD grade 3 or above	0.70	0.46–1.05	0.085
ICU stay	0.92	0.60–1.43	0.738
Prophylactic anticoagulation a	0.29	0.15–0.58	<0.001
Therapeutic anticoagulation a	0.15	0.07–0.32	<0.001
CS treatment duration (day)	0.89 b	0.84–0.93	<0.001

Abbreviations: CKD, chronic kidney disease; CS, corticosteroid; ICU, intensive care unit.

a Compared with No anticoagulation.

b Per 1-day increase.

Results of four variations of the same model with single-variable changes are shown in Table 3 . When AC duration replaced the categorical variable (tAC, pAC, and no AC), the number of days on AC was an important predictor of longer time to death (HR = 0.89 per 1-day increase; 95% CI: 0.85–0.94; *p* < 0.001). The timing of tAC (first 1–2 days of hospitalization vs. day 3 and beyond) was also explored in multivariate analysis and earlier initiation of tAC did not appear to provide the same benefit (HR = 0.63; 95% CI: 0.38–1.01; *p* = 0.055) compared with later initiation (HR = 0.32; 95% CI: 0.19–0.55; *p* < 0.001). As a binary variable, the effect of CS treatment remained significant (HR = 0.62; 95% CI: 0.43–0.91; *p* = 0.016). Initiation of CS treatment on days 1 to 2 of hospitalization (HR = 1.41; 95% CI: 0.84–2.30; *p* = 0.187) had no effect on time to death, whereas initiation on day 3 and later showed a decrease in risk of death (HR = 0.48; 95% CI: 0.28–0.68; *p* = 0.001). In all variant models, other variables from the original model had similar effects.

**Table 3:** Variant multivariate Cox proportional hazards models

		Hazard ratio	Confidence interval	Significance
1	AC treatment duration	0.89 a	0.85–0.94	<0.001
2	AC started day 1–2	0.63	0.38–1.01	0.055
	AC started day ≥3	0.32	0.19–0.55	<0.001
3	CS treatment b	0.62	0.43–0.91	0.016
4	CS started day 1–2	1.41	0.84–2.30	0.187
	CS started day ≥3	0.48	0.28–0.68	<0.001

Abbreviations: AC, anticoagulation; CS, corticosteroid.

a Per 1-day increase.

b Compared to No CS treatment.

In analyses conducted separately on severe (no ICU stay, *n* = 52) and critical disease (required ICU stay, *n* = 75), the impact of tAC and pAC remained significant with a larger effect size in delaying death in the critical COVID-19 population ( Supplementary Tables S1 and S2 ).

No interactions between considered variables were noted; furthermore, there was no interaction between tAC and D-dimer ( *p* = 0.260) in a model including only patients for whom the latter was known ( *n* = 94).

## Discussion

To the best of our knowledge, ours is among the first studies to investigate the impact of therapeutic AC on clinical outcomes of severe or critical COVID-19 patients. Our main findings are as follows: (1) AC delayed death in a dose- and duration-dependent manner; (2) CS treatment delayed death in a duration-dependent manner; and (3) the rate of bleeding was not significantly higher for patients treated with AC, but there are concerns about increased infectious complications in patients treated with CS.

In our study population, nearly all patients (90%) received some form of AC and more than half received therapeutic doses. The most frequently used anticoagulant was intravenous unfractionated heparin (87%), which was likely preferred for its superior safety profile, especially in the setting of renal failure. Over half of patients who received tAC did so empirically to treat COVID-19-associated hypercoagulability. It is unknown how many of these patients had unidentified venous thromboembolic disease as the use of diagnostic imaging was severely limited.

The optimal time, dosing, and duration of tAC are unknown. For most patients, AC treatment was initiated relatively late in the hospital course (median of day 6) as a consequence of rapidly changing practice patterns as new data becomes available. Our analysis suggests that timing of tAC may be important. Specifically, later initiation of tAC (day 3 of hospitalization and later) appears to have a higher impact. This observation highlights that tAC is likely more beneficial in a later phase of COVID-19 when severe inflammation and activation of the coagulation cascade play the major role in pathogenesis.

The median duration of AC was 5 days. Our data demonstrated an increase in time to death with increasing duration of tAC (∼10% decrease in the risk of death per 1-day increase of tAC duration).

Most striking, however, was the increasing impact on time to death with increasing doses of AC. Compared with no AC, the use of prophylactic doses decreased the risk of dying by over 70% and therapeutic doses by 85%. When factored in as duration of treatment, tAC showed a decrease in the risk of death at any time point (HR = 0.89 per 1-day increase; 95% CI: 0.85–0.94; *p* < 0.001). These effects were independent of other life-support modalities (vasopressor use, mechanical ventilation, and renal replacement therapy) and CS treatment. Our findings are consistent with those reported by Tang et al, 13 whose retrospective analysis found that pAC decreased 28-day mortality in a subgroup of patients with SIC score ≥ 4 (40 vs. 64.2%; *p* = 0.029) or with D-dimer > 6-fold the upper limit of normal (32.8 vs. 52.4%; *p* = 0.017). Our data showed a difference in time to death regardless of D-dimer level, but it is important to note that pAC was employed in nearly all patients and tAC was preferentially initiated per institutional criteria based on the Tang et al study in the presence of D-dimer > 6-fold the upper limit of normal. In a recently published research letter, Paranjpe et al 14 analyzed the impact of tAC in a large cohort of COVID-19 patients. In-hospital mortality was similar in those who received tAC and those who did not (22.5 vs. 22.8%), but median survival time was significantly better in the AC group (21 vs. 14 days). A much larger benefit for tAC was observed in mechanically ventilated patients, who had lower in-hospital mortality (29.1 vs. 62.7%) and improved median survival (21 vs. 9 days). In a multivariate model, the duration of tAC was independently associated with a decrease in the risk of death at any time point (HR = 0.86 per 1-day increase; 95% CI: 0.82–0.89; *p* < 0.001), which was similar in effect size to the one observed in our population (HR = 0.89). It is unclear if the patients in the analysis by Paranjpe et al who did not receive tAC received any form of pAC. The authors acknowledge several important limitations such as the observational nature of the study, hidden confounding, and difficulties with classification of disease severity. By focusing on deceased patients only, our analysis hopes to, at minimum, avoid confounding by indication. Although it remains difficult to extrapolate what the true, prospective benefit of tAC is in a population that has had time to complete the disease course, our findings complement and strengthen those of Paranjpe et al.

We agree with the reservations about the use of tAC in severe COVID-19 voiced by professional societies, 4 16 given the potential for serious adverse events, chiefly bleeding. It is important to note, however, that in our cohort the rates of all types of bleeding were similar among those who received tAC and those who did not, suggesting that this therapeutic strategy can be relatively safe. Similarly, Paranjpe et al found no significant increase in bleeding events with tAC (3 vs. 1.9%, *p* = 0.02) and reported that more than half the bleeding events in the tAC group occurred before initiation of AC.

An additional interesting observation was the effect of CS treatment in increasing time to death. At least one prior retrospective analysis 20 has reported a mortality benefit with methylprednisolone treatment in COVID-19 patients with acute respiratory distress syndrome (62% decrease in univariate analysis). Nonetheless, experience with CS in the treatment of acute respiratory distress syndrome caused by related viruses SARS-CoV-1 and Middle east respiratory syndrome (MERS) was associated with frequent side effects without any observed clinical benefit. 21 22 Furthermore, quality data have demonstrated increased mortality in influenza pneumonia treated with CS. 23

With the above caveats in mind, we note that in our population, the use of CS showed a 44% decrease in the risk of death in univariate analysis (comparable to 20 ) and a more modest 38% decrease in multivariate analysis. Results suggested that CS treatment duration (median = 5 days, consistent with institutional guidelines) may also be important, and we observed an 11% reduction in risk of death per day of treatment. Once again, this data should be interpreted with great caution. We did notice an increased rate of infectious complications among patients treated with CS (13–18 vs. 8% positive blood or respiratory cultures). Similar to tAC, CS treatment had a stronger effect when initiated later during hospitalization. However, in contrast to criteria for tAC, institutional guidelines do not clearly define when CS are warranted and the decision is made primarily by clinical impression. It is likely that recognition of more severe disease resulted in earlier initiation of CS.

### Limitations

This cross-sectional, observational study has several important limitations and we advise caution in interpreting results. Beyond inherent bias stemming from hidden confounders, our selection of only deceased patients makes it impossible to derive conclusions about the effect of any therapeutic modality on long-term survival. By design, the study can only report associations and cannot investigate causality. Furthermore, the population that was available for analysis was inhomogeneous in terms of practice, as institutional guidelines were adopted halfway through the study period and have resulted in significant practice changes.

Despite these limitations, the study offers new and urgently needed data on the effect of AC in the treatment of severe COVID-19, a disease that suffers from a serious lack of available therapeutic modalities of proven efficacy. Ours represents an interim analysis of prospective data that aim to further investigate the effect of AC and immunosuppression on outcomes in COVID-19.

## Conclusion

Activation of the coagulation cascade resulting in a hypercoagulable state with subsequent visceral microthrombosis is increasingly recognized as a hallmark of the pathogenesis of COVID-19. The impact of AC on delaying death in severe COVID-19 appears to be dose- and duration-dependent, with greater effect seen for therapeutic compared with prophylactic doses. Immunosuppressive doses of CS also delayed death to a more modest extent. Despite important limitations, our findings support those of others who have reported a survival advantage with prophylactic and therapeutic AC in this population. Although, bleeding complications were similar to nonanticoagulated patients, the decision to initiate AC should remain individualized and always take into consideration of the individual risk of bleeding. Despite seemingly encouraging results for CS, the rate of infectious complications was higher. We recognize the urgent need for further randomized controlled trials to explore the therapeutic effects of tAC and CS in critically ill patients with COVID-19.
