# Supplementary material for: Therapeutic Anticoagulation Delays Death in COVID-19 Patients: Cross-Sectional Analysis of a Prospective Cohort
Source: TH Open. 2020 Sep 26;4(3):e263–70. doi: 10.1055/s-0040-1716721 (PMC7519875; doi:10.1055/s-0040-1716721)
Supplement: Supplementary file 1 — Supplementary Material [file 10-1055-s-0040-1716721-s200040.pdf]

Supplementary Table S1 Multivariate Cox proportional hazards model (severe disease, $n = 52$)

	Hazard ratio	Confidence interval	Significance
Ever smoker	1.36	0.75–2.48	0.308
CKD grade 3 or above	0.67	0.32–1.33	0.266
Prophylactic anticoagulationc	0.36	0.16–0.8	**0.014**
Therapeutic anticoagulationc	0.27	0.08–0.55	**0.001**
CS treatment duration (days)	0.87[a]	0.76–0.98	**0.033**

Abbreviations: CKD, chronic kidney disease; ICU, intensive care unit; CS, corticosteroid.
Note: Bold represents statistically significant values.
[a]Per 1-day increase.
[b]Compared to No anticoagulation.

Supplementary Table S2 Multivariate Cox proportional hazards model (critical disease, $n = 75$)

	Hazard ratio	Confidence interval	Significance
Ever smoker	2.20	1.33–3.66	0.002
CKD grade 3 or above	0.70	0.41–1.17	0.18
Prophylactic anticoagulationc	0.15	0.04–0.73	**0.022**
Therapeutic anticoagulationc	0.07	0.02–0.32	**0.002**
CS treatment duration (days)	0.89[a]	0.84–0.95	**0.001**

Abbreviations: CKD, chronic kidney disease; ICU, intensive care unit; CS, corticosteroid.
Note: Bold represents statistically significant values.
[a]Per 1-day increase.
[b]Compared to No anticoagulation.
